# The Hardiness of Adolescents in Various Social Groups

**DOI:** 10.3389/fpsyg.2019.02427

**Published:** 2019-10-25

**Authors:** Valerii Malkin, Liudmila Rogaleva, Alla Kim, Natalya Khon

**Affiliations:** ^1^Department of Theory of Physical Culture, Ural Federal University, Yekaterinburg, Russia; ^2^Department of General and Applied Psychology, Al-Farabi Kazakh National University, Almaty, Kazakhstan; ^3^Department of Psychology, Turan University, Almaty, Kazakhstan

**Keywords:** hardiness, adolescents, social environment, activities, development

## Abstract

Hardiness is considered as one of the adaptation resources of personality to stressful situations, responsible for the mental health preservation. Adolescents as an age group is one of most susceptible to stress factors, so conducting research on the development of hardiness in adolescents becomes necessary. Due to difference in social conditions under which development of hardiness of adolescents takes place, the purpose of our research was to study the hardiness of adolescents included in different social groups: first group – athletes, students of sports schools, second – students of specialized schools for intellectually gifted individuals, third– students of regular schools. In total, 239 adolescents of 14–16 years old participated. The study revealed significant differences in the development of hardiness among all three groups of adolescents. The general and specific patterns of the development of hardiness components of adolescents in different social groups were identified. The article describes the main types of manifestation of hardiness and its components under different social conditions of activity and development in modern adolescents.

## Introduction

The problem of adaptation to the requirements of the social environment is particularly acute in adolescence, which is traditionally regarded as a crisis age (Kalashnikova and Petrova, 2017; Benzi et al., 2019; Morinaj and Hascher, 2019). The desire of adolescents to experience their own strength, to succeed and to assert themselves in the peer group is associated with various kinds of difficulties that can lead to inappropriate forms of behavior (Sobkin et al., 2005; Echazu and Nocetti, 2018). Because of this, more and more research is connected with the study of factors that ensure the development of hardiness, which is considered as the main adaptation resource of a person responsible for preserving the mental health and well-being of adolescents (Kiva et al., 2016; Oschepkov, 2017; Mishina, 2018).

According to the studied literature, various interrelated factors (genetic, social, age) of the controversial development of adolescents’ hardiness are distinguished (Ivanova, 2013; Kormushina, 2016; Gorkovaya and Miklyaeva, 2017; Bezgodova et al., 2018). At the same time, most authors believe that the development of the hardiness of adolescents is determined to a greater degree by the social environment (Shvareva, 2010; Gorkovaya et al., 2015; Casagrande et al., 2018; Fokina et al., 2018; Guerra et al., 2019, etc.).

This is confirmed by a number of studies that prove the positive or negative impact of various social groups on the development of hardiness (Borzilova and Solonchenko, 2017; Kalashnikova and Nikitina, 2017; Nikitina, 2017; Melisbek and Bodnar, 2018). In particular, it proves the positive role of a happy family in the development of hardiness compared with the unfavorable one (Archakova, 2016; Gulyaeva and Myagkaya, 2017).

In a number of works it has been revealed that hardiness is more pronounced among adolescents involved in sports, creative, intellectual, or vocal activities, compared to ordinary schoolchildren (Sultanbaeva and Chertykov, 2014; Chernyavskaya and Shabanova, 2016; Plotnikov et al., 2017; Shumakova and Shamardina, 2018). Without disputing the significance of these studies it should be noted that they were conducted on a small sample and are rather local.

We believe that to study the role of the influence of the social environment on the development of the hardiness of adolescents, it is necessary, first of all, to determine the methodological position. In our opinion, this approach can be the social activity approach of the Russian psychologists – classics such as Ananyev (2007) and Rubinstein (2015), as well as the works of foreign researchers – Deci and Ryan (2000), Bandura (2004), and Seligman (2013).

The essence of this approach is that the influence of the social environment on the development of adolescents is always mediated by the type of activity that is leading for them and the conditions in which this activity is carried out. In the course of mastering activities and adapting to social conditions adolescents will develop the qualities that will be more necessary for carrying out activities and adapting to a specific environment. This methodological approach was the basis of our research presented in this article.

In determining hardiness, we rely on the work of Kobasa (1979) and Leontiev and Rasskazova (2006), who understand hardiness as the measure of personality’s ability to withstand a stressful situation, maintaining internal balance without reducing success. It includes three autonomous components: commitment, control and challenge.

Commitment includes interest in their activities. The opposite phenomenon is detachment from social interests and life in general. Control implies the presence of their own beliefs, ideas, even in case of unwarranted success. The opposite phenomenon of control is helplessness, inability to defend one’s opinion, to deny or insist on it. Challenge is based on a person’s ability to be aware of his/her actions and decisions, being aware of the degree of risk in a given case at the same time (Leontiev and Rasskazova, 2006).

**FIGURE 1:**
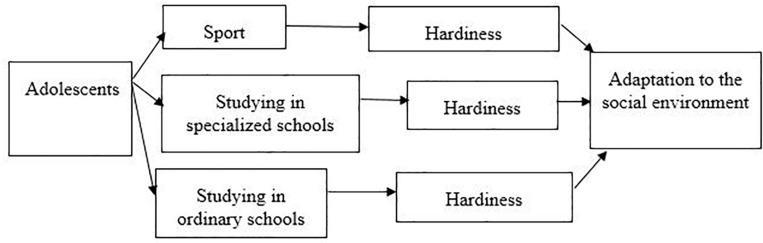
Model of the influence of the social environment on the development of hardiness of adolescents.

Based on the foregoing, it is possible to consider the above presented approach in the form of a scheme, according to which the development of the hardiness of adolescents to a greater extent is determined by activity (Figure 1).

The methodological basis of the study allowed us to identify three social groups of adolescents, who, due to a certain specificity of the performing activity may differ in terms of the level of development and components of hardiness. The number of such groups included adolescents involved in sports schools and schools of the Olympic reserve, making up the first group, students of gymnasiums, lyceums as the second group, students of secondary schools made up the third group.

The specificity of each group is determined by the dominant orientation of the activity in which they are included. In the first and second group there were either achievements in sports activities or academic achievements, in the third group students were more focused on their individual needs. The purpose of the study was to identify the similarities and differences, the influence of various social environments on the development of hardiness of adolescents and its components (commitment, control, and challenge).

### General Hypothesis

It is assumed that the inclusion of adolescents in different social conditions of life can affect the development of their level and components of hardiness (commitment, control, challenge).

It is assumed that obtaining objective knowledge on the impact of social conditions and activities on the development of hardiness may be helpful for the development of more effective strategies for working with adolescents and improving their well-being and mental health.

### Particular Hypotheses

Sporting activity takes place in conditions of real competition, aiming at both immediate and distant goals, therefore it is associated with stress-competitive situations, with the need to overcome difficulties, so it can be assumed that the existence of hardiness, on the one hand, is one of their main conditions of success, and therefore all components of hardiness are of high importance and must be developed at a sufficiently high level.

Learning activity in gymnasiums, lyceums and specialized research centers is focused on high academic achievements with orientation on future perspectives, it seems to be impossible without self-control, and therefore we can assume that this component must be high.

At regular school adolescents are in more natural conditions of life, they are more left to themselves, have fewer commitments and more free time to meet their individual needs. At the same time lack of sufficiently clear and long-term goals, peculiar for the other two groups of adolescents, will affect the development of hardiness components. So, the specificity of each group is determined by the dominant orientation of the activity in which they are included. In the first group these are sports results, in the second group the main is orientation is academic knowledge, while in the third group students are more focused on meeting their individual needs.

## Materials and Methods

### Research Design

The study was conducted on the basis of the Olympic Reserve School, the “Yunost” Children and Youth Sports School, the Specialized Educational and Specialized Educational and Scientific Center (lyceum) for Gifted Schoolchildren, the Gymnasium with in-depth study of English and several secondary schools in Yekaterinburg. The research Ethics Committee of the Ural Federal University of Yekaterinburg approved this study. Written informed consent was obtained from the parents/legal guardians of all participants. The distribution of participants by gender is, in our opinion, very important in terms of its significance for determining behavior and readiness to overcome stress in the social environment (Gorkovaya et al., 2015; Kalashnikova and Petrova, 2017). The study involved 239 adolescents aged from 14 to 16 years, 80 adolescents-sportsmen (44 male and 36 female), 79 adolescents – lyceum students (39 male and 40 female), 80 adolescents- schoolchildren (37 male and 43 female).

### Measures and Statistical Analysis

As a research method, we selected testing of adolescents from different social groups of 14–16 years old. To study hardiness, we used a questionnaire developed by the American psychologist Salvatore Maddy and adapted by Leontiev and Rasskazova (2006). The questionnaire consists of 45 questions, allow you to assess the level of hardiness, as well as indicators of 3 subscales (commitment, control, and challenge). The reliability determined for this investigation was α = 0.90.

To compare hardiness indicators the SPSS-23 program (Students *t*-test and the Kruskal–Wallis test) was used.

## Results

In the course of the study, we identified differences in the level of development of hardiness in adolescents of different social groups. The levels of development of resilience were determined in accordance with the data from the Leontiev and Rasskazova (2006), according to which the low level is less than 60 points, the average level is between 60 and 90 points, and the high level is above 90 points. The results of the study are presented in Table 1.

**TABLE 1:** The level of development of hardiness of adolescents of different social groups.

**Samples parameters**	**Number of participants with different level of hardiness (%)**		
	
	**Low level**	**Middle level**	**High level**
For the sample as whole	14.2%	52.9%	32.9%
Adolescents girls	20.3%	54.3%	25.4%
Adolescents boys	7.3%	52.6%	40.1%
Adolescents- sportsmen	2%	53,5%	44.5%
Adolescents Lyceum students	15%	60%	25%
Adolescents-schoolchildren	23%	46%	31%
Boys athletes	2.2%	55.6%	42.2%
Lyceum students boys	5.1%	66.7%	28.2%
School boys	16.2%	32.5%	51.3%
Girls athletes	2.7%	54%	43.3%
Lyceum students girls	25%	52.5%	22.5%
Schoolgirls	31.7%	56.3%	12%

According to the data in Table 1, it is possible to note the differences in the indices of the development of hardiness both between three social groups and in the gender indicators within each group. First of all, talking about each group as a whole, we can conclude that 44.5% of adolescent -sportsmen have high level of hardiness, and only 2% have low rates. At the same time, if we talk about gender indicators, then indicators of the level of development of hardiness are almost the same in girls and boys.

The data obtained show that the competitive environment with a focus on achievement and a specific result, ensures the development of hardiness of adolescent- sportsmen at a sufficiently high level. In the group of lyceum students and schoolchildren the data obtained show similar to adolescent- sportsmen average indicators of hardiness. Number of adolescents with a high level (25%) is reduced and there is a clear increase in adolescents with a low level (15%). Moreover, the low values of hardiness are more pronounced in female lyceum students.

The analysis of the questionnaires of schoolchildren with high indicators of hardiness revealed interesting facts, in particular, young students of physical and mathematical classes, winners of scientific competitions, and young men of gymnasiums, but successfully combining sports with educational achievements, have high rates.

Thus, the findings confirm the fact that the competitive environment and focus on future achievements will increasingly develop the hardiness of adolescents. Relating the difficulties of adaptation in the gender aspect, it is obvious that in the academic environment girls find it harder to adapt.

There is a significant difference in the manifestation of hardiness in the two groups: only 22.5% of lyceum girls’ students have high values vs. 43.3% in female athletes, 25% of them have low values vs. 2.7% in female athletes.

Thus, it can be concluded that the development of hardiness in the groups of adolescents of lyceum students differs from the group of adolescents-sportsmen.

In the third social group of adolescents –schoolchildren, the peculiarities of manifestation of hardiness are also noted, compared with the first and second groups. First, the level of high development of hardiness in this sample is lower (31%) than in the sample of adolescents sportsmen (44.5%), but slightly higher than that of adolescents lyceum students (25%).

At the same time, these high values are provided primarily by data on boys schoolchildren (51, 3%), which is higher than in other groups. The data obtained can be explained on the basis of questionnaires of adolescents with high rates, so they all have hobbies, in particular, go in for sports (recreational sports), but for them it’s more a type of free leisure, not a focus on results, and secondly, they are quite independent in choosing activities and achieving their short-term goals, thirdly, they do not bear the burden of high responsibility imposed by adults.

Along with the pluses, we see that the number of adolescents schoolchildren with low values is also quite large (23%). This means that a fairly large group of adolescent schoolchildren do not develop hardiness, most likely due to the fact that these students are not involved in active forms of creative and sports activities. Low values throughout the sample were observed in 13.5% of adolescents, while it can be noted that the number of girls with a low level of hardiness exceeds boys, respectively 20, 3% and 7, 3%.

The group with low values includes not only girls schoolchildren (31.7%), but also boys schoolchildren (16.2%), and this means that a fairly large group of adolescents schoolchildren does not develop hardiness due to the fact that there are no conditions for its manifestation, these schoolchildren are not involved in active forms of creative and sports activities. Thus, the obtained data confirm the fact that a competitive environment and a focus on sporting achievements will contribute more to the development of the hardiness of adolescents than the academic environment focused on academic achievements, even if they are quite high. Thus, it can be concluded that all social groups have conditions for the development of the hardiness of adolescents, but it is the sports environment that has the most significant influence on its development. The presented results can be supplemented with data on the study of the development of components of hardiness, such as commitment, control, and challenge. To identify differences in the development of components of hardiness among adolescents from different social groups, mathematical data processing methods were used.

Analysis of the development of components of hardiness in the groups of adolescents – sportsmen and adolescents – lyceum students, presented in Table 2, suggests that there are significant differences in the component of commitment and general hardiness in these groups.

**TABLE 2:** Data on hardiness and its components (commitment, control, challenge) in the group of adolescents-sportsmen and adolescents-lyceum students.

	**Mean value**		**Standard deviation**			
				
	**Adolescents-sportsmen**	**Adolescents-lyceum students**	**Adolescents-sportsmen**	**Adolescents-lyceum students**	***t***	**Cohen coefficient**
Commitment	35,4875	29,0127	7,23388	7,70530	5,461^∗∗^	0.86
Control	33,0000	31,4684	7,30528	8,12693	1,250	0.27
Challenge	18,7375	17,4177	3,94180	5,17293	1,811	0.24
Hardiness	87,0250	77,5063	15,38862	18,34560	3,542^∗∗^	0.60

*^∗^*p* < 0.05; ^∗∗^*p* < 0.01.*

There are no significant differences in the components of control and challenge, this fact indicates that academic activities, as well as sports activities, contribute to the development of these components of hardiness. Cohen coefficient shows high effect for commitment (0.86) and middle effect for hardiness (0.60).

We can see in Table 3 significant differences in control and challenge components in the studied groups. The indicator of hardiness, “control,” is the conviction of adolescents that their efforts depend on personal results and they can influence the outcome of what is happening, even if this influence is not absolute and success is not guaranteed. Adolescents with a developed component of control feel that they choose their own activity, their own way. Thus, this suggests that in the framework of sports and academic activities, the development of control and challenge is higher than in the framework of general academic activities, whereby the development of hardiness is higher among adolescents-sportsmen and adolescents-lyceum students. Most likely, this is due to the fact that competition and a focus on achieving high sports or academic results require a greater manifestation of effort and readiness to work in conditions of both success and non-success. Cohen coefficient shows middle effect for commitment and control (0.53, 0.44) and high effect for challenge (0.88).

**TABLE 3:** Data on hardiness and its components (commitment, control, challenge) in the group of adolescents lyceum students and adolescents schoolchildren.

	**Mean value**		**Standard deviation**			
				
	**Adolescents-lyceyists**	**Adolescents-schoolchildren**	**Adolescents-lyceyists**	**Adolescents-schoolchildren**	***t***	**Cohen coefficient**
Commitment	29,0127	33,3500	7,70530	8,73535	−3,321^∗∗^	0.53
Control	31,4684	27,8500	8,12693	10,43254	2,441^∗∗^	0.44
Challenge	17,4177	13,2125	5,17293	4,68959	5,368^∗∗^	0.88
Hardiness	77,5063	74,4625	18,34560	19,97020	1,001	0.16

*^∗^*p* < 0.05; ^∗∗^*p* < 0.01.*

The obtained data confirmed the hypothesis put forward by us that in conditions of sports activity in adolescents the level of development of hardiness is higher than that of adolescents lyceum students and adolescents schoolchildren. At the same time, it can be noted that the influence of the academic environment and educational activities on the development of control and challenge in adolescents is quite high. Regarding adolescent schoolchildren, it can be said that in this environment there are conditions for developing the hardiness of adolescents, but only if they are included in active forms of leisure (sports, social, and creative groups).

Significant gender differences in commitment, control and general hardiness have been revealed. Boys in general have priority in Table 4. It can be concluded that it is the academic environment that is less conducive to the development of the hardiness of girls, while in the sports environment conditions are almost equal. Cohen coefficient shows middle effect for commitment and control (0.48, 0.50) and high effect for hardiness (0.59).

**TABLE 4:** Gender differences on hardiness and its components (commitment, control, challenge).

	**Mean value**		**Standard deviation**			**Cohen coefficient**
				
	**Boys**	**Girls**	**Boys**	**Girls**	***t***	
Commitment	33,9832	31,2231	8,15733	8,31914	2,595^∗∗^	0.48
Control	32,6050	28,8926	8,20316	9,30663	3,276^∗∗^	0.50
Challenge	16,8487	16,0000	5,31490	5,05140	1,268	0.24
Hardiness	83,7479	75,4545	17,82695	18,78874	3,507^∗∗^	0.59

*^∗^*p* < 0.05; ^∗∗^*p* < 0.01.*

In fact, our data confirm studies indicating the important role of the social environment in the development of endurance of adolescents (Sultanbaeva and Chertykov, 2014; Archakova, 2016; Chernyavskaya and Shabanova, 2016; Malkin and Rogaleva, 2016; Borzilova and Solonchenko, 2017; Gulyaeva and Myagkaya, 2017; Plotnikov et al., 2017; Shumakova and Shamardina, 2018; Straub, 2019).

In our opinion, this can be explained that different types of activities do not equally affect the satisfaction of the needs for autonomy, competence and for relatedness (Deci and Ryan, 2000) and the achievement of self-efficacy (Bandura, 2004; Rubinstein, 2015; Nikitina, 2018; Rogaleva et al., 2018).

The data obtained can be used to develop strategies for educational programs for the correction of social conditions for the development of hardiness in adolescents of different social groups, ensuring their well-being. In particular, for adolescents of all social groups, one of the main conditions for increasing hardiness is the presence of involvement and interest in the implementation of activities or social interaction.

The second important aspect is the need to set short and long-range goals, which is typical for sports activities, and academic orientation schools and that are not sufficiently present in regular schools. Target attitudes and action to achieve them, can contribute to the development of self-control, risk-taking, acceptance of success and failure as an important experience that can increase adolescent confidence.

The third important component of the strategy for working with adolescents is to develop risk preparedness or the desire to actively acquire knowledge from experience for later use, this is achieved only if the substantive component of programs provides for linking activities with future professional activities, or with those skills which may be useful in mastering a particular profession, or have some real practical result that a teenager can be proud of and perceive it as kind of achievement significant for the future.

## Conclusion

Thus, our study confirms the hypotheses put forward, according to which the development of the hardiness of adolescents depends on the activity they perform and the conditions in which the activity is carried out. The obtained new data can contribute to the construction of a strategy for working with adolescents and various social groups to achieve their well-being. In this regard, further studies may be aimed at studying the hardiness of adolescents in the context of availability of conditions for satisfying their needs in autonomy, competence and significant relationships, which are considered as factors of their well-being.

## Data Availability Statement

All datasets generated for this study are included in the article/supplementary material.

## Ethics Statement

All procedures performed in studies involving human participants were in accordance with the ethical standards of the institutional and/or national research committee and with the 1964 Helsinki Declaration and its later amendments or comparable ethical standards.

## Author Contributions

VM, LR, AK, and NK conceived the hypothesis of this study, and wrote the manuscript with significant input from VM. VM and LR participated in data collection. All authors analyzed the data, contributed to data interpretation of the statistical analysis, and read and approved the final manuscript.

## Conflict of Interest

The authors declare that the research was conducted in the absence of any commercial or financial relationships that could be construed as a potential conflict of interest.
